# Menopause-associated gut microbiome remodeling and pharmacomicrobiomics: emerging mechanisms influencing drug response

**DOI:** 10.3389/frmbi.2026.1953547

**Published:** 2026-09-15

**Authors:** Susanne B. Haga

**Affiliations:** Division of General Internal Medicine, Department of Medicine, Duke University School of Medicine, Durham, NC, United States

**Keywords:** drug response, endocrinology, gut microbiome, menopause, pharmacomicrobiomics

## Abstract

Menopause is accompanied by profound hormonal changes that alter the composition and function of the gut microbiome, potentially influencing microbial drug metabolism and therapeutic response. At the same time, medication use increases substantially during midlife, creating a dynamic bidirectional relationship in which medications modify the gut microbiome while microbiome remodeling may influence medication efficacy, toxicity, and interindividual differences in drug response. This narrative review examines current evidence linking menopause-associated changes in the gut microbiome with medication response and explores the biological mechanisms that may underlie these interactions. The review summarizes microbiome-mediated pathways including microbial β-glucuronidase activity, direct microbial drug metabolism, altered bile acid metabolism, microbial metabolite signaling, and changes in intestinal barrier function and immune regulation that may influence pharmacokinetics and pharmacodynamics. Although direct evidence connecting menopause-associated microbiome remodeling with altered drug response remains limited, substantial evidence supports independent effects of menopause on the gut microbiome and of the gut microbiome on drug metabolism. Together, these findings provide a conceptual framework for understanding how menopause-associated microbial changes may contribute to variability in pharmacotherapy and highlight important priorities for future research, including incorporation of menopausal status into pharmacokinetic studies and clinical trials to advance microbiome-informed precision medicine for women.

## 1 Introduction

Over the past decade, interest in menopause and the human microbiome has expanded rapidly, reflecting growing recognition of their important roles in health and disease, respectively. Although these fields have traditionally been studied independently, accumulating evidence suggests that they intersect in clinically meaningful ways, creating a unique biologic context in which gut microbiome-mediated variability in drug response may become increasingly important (Younes et al., 2018). This relationship is particularly relevant because the menopausal transition coincides with increasing chronic disease burden (Boersma et al., 2020; Kamińska et al., 2023) and prescription medication use (Charlesworth et al., 2015; Sutherland et al., 2015; Martin et al., 2019; Wang et al., 2023b; Miao et al., 2025). As illustrated in **Figure 1**, the menopausal transition represents the convergence of declining estrogen concentrations, increasing chronic disease burden, rising medication use, and alterations in gut microbial composition and function. Together, these changes create a biologically plausible framework through which menopause-associated microbiome alterations may contribute to variability in drug response.

**Figure 1:**
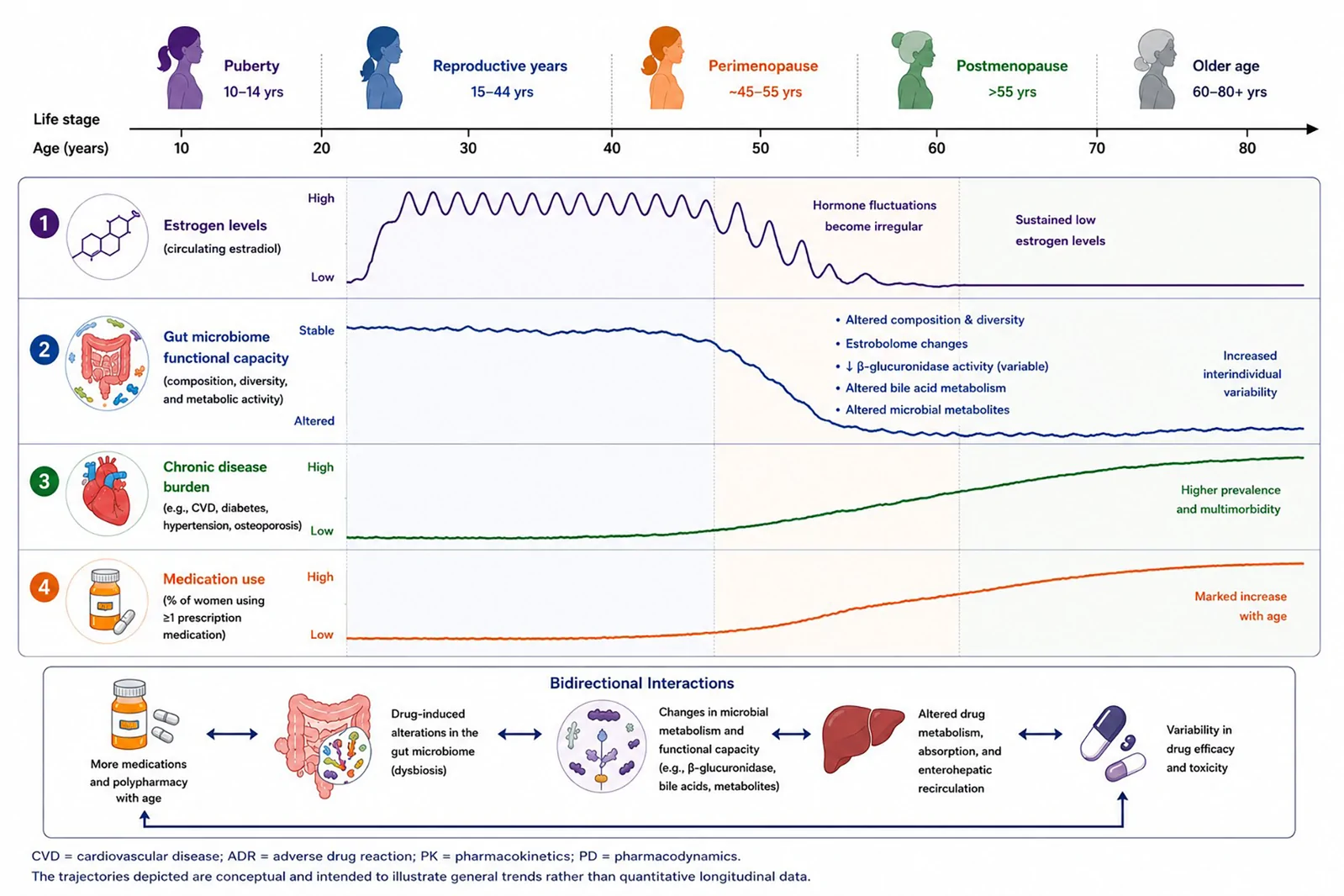
Conceptual framework linking menopause, gut microbiome remodeling, and variability in drug response. Declining estrogen concentrations during the menopausal transition coincide with increasing chronic disease burden, greater medication use, and alterations in gut microbial composition and functional capacity. Together, these age-related changes create a biologically plausible framework in which menopause-associated gut microbiome remodeling may influence drug disposition, therapeutic efficacy, and adverse drug reactions. Although direct clinical evidence remains limited, the convergence of endocrine, microbial, and pharmacologic changes suggests that menopause represents a critical life stage for investigating microbiome-mediated variability in drug response.

The gut microbiome is increasingly recognized as an important modulator of drug disposition and therapeutic response (Sousa et al., 2008; Noh et al., 2017; Zhang et al., 2018; Zimmermann et al., 2019; Pant et al., 2023; Zhao et al., 2023). Through a variety of mechanisms, including direct microbial metabolism of medications, modulation of drug absorption and intestinal barrier function, alteration of enterohepatic circulation, production of bioactive metabolites, and regulation of host immune and metabolic pathways, the microbiome can influence both the efficacy and toxicity of numerous therapeutic agents. This emerging field, known as *pharmacomicrobiomics*, has highlighted the microbiome as an important contributor to variability in pharmacokinetics and pharmacodynamics that complements traditional determinants of drug response, including host genetics, age, organ function, comorbidities, and concomitant medications (Gronich et al., 2025).

The menopausal transition is accompanied by profound endocrine changes, particularly declining estrogen levels, that are associated with alterations in the composition and function of the gut microbiome (Saravinovska et al., 2026). Changes in microbial metabolic function may influence drug metabolism, absorption, and enterohepatic circulation. However, limited research has been published about how menopause-associated microbial changes may influence drug metabolism, therapeutic effectiveness, and adverse drug reactions.

Given the increasing number of women living for decades post-menopause and the high prevalence of polypharmacy in this age group, understanding the interactions among menopause, the microbiome, and medication response has important implications for precision medicine. Understanding these relationships may improve precision pharmacotherapy for midlife and older women.

This narrative review synthesizes current evidence regarding estrogen-associated changes in the gut microbiome across a woman’s lifespan, with particular emphasis on the menopausal transition, and examines the mechanisms through which these microbial alterations may influence drug disposition, efficacy, and toxicity. Although the gut microbiome comprises bacteria, viruses, fungi, archaea, and their collective genetic and metabolic functions, the available literature on menopause-associated microbiome changes and drug response has focused predominantly on bacterial communities; accordingly, these constitute the primary focus of this review. While menopause-associated alterations in gut microbial composition and function are now increasingly recognized, uncertainty remains regarding the extent to which these changes translate into clinically meaningful differences in drug response. Current knowledge gaps and future research priorities are discussed to advance integration of the gut microbiome into precision pharmacotherapy for midlife and older women.

## 2 Estrogen changes across the female lifespan

Over the female lifespan, estrogen concentrations fluctuate substantially during puberty, pregnancy, the menopausal transition, and postmenopause (Frederiksen et al., 2020; Santoro et al., 2021; Patel et al., 2025). Puberty and pregnancy are characterized by rising estrogen concentrations, whereas menopause is marked by a sustained decline following ovarian failure. These hormonal transitions provide the biologic context for understanding subsequent alterations in the gut microbiome and their potential implications for drug response.

## 3 Bidirectional interactions between estrogen and the gut microbiome

The relationship between estrogen and the gut microbiome is increasingly recognized as bidirectional, with each influencing the composition, function, and metabolic activity of the other. This reciprocal communication forms the basis of the host–microbiome endocrine axis and provides the biologic framework through which menopause-associated hormonal changes may influence both health and drug response (Clemente-Suárez et al., 2024; Mou et al., 2026).

Experimental and clinical studies demonstrate that estrogen influences both the composition and functional capacity of the gut microbiome (Baker et al., 2017; Peters et al., 2022; Saravinovska et al., 2026). Estrogen receptors are expressed throughout the gastrointestinal tract, where estrogen contributes to the regulation of epithelial barrier integrity, mucosal immunity, intestinal permeability, inflammatory signaling, and bile acid metabolism (Baker et al., 2017; Kumari et al., 2024). Estrogen also influences bile acid synthesis and composition, which in turn affects the intestinal environment and microbial ecology (Fiorucci and Distrutti, 2015; Wahlström et al., 2016). These physiologic effects help shape the intestinal environment and influence microbial composition and function. Consequently, declining estrogen during menopause has been associated with alterations in gut, vaginal, oral, and urogenital microbial communities (Tramice et al., 2022; Park et al., 2023).

Alterations in gut microbial composition and function associated with declining estrogen have been implicated in the pathogenesis of numerous health conditions, including metabolic dysfunction, osteoporosis, depression, cardiovascular disease, and hormone-related cancers (de Kruif et al., 2016; Kwa et al., 2016; Chen et al., 2017; Ryczkowska et al., 2023; Wang et al., 2023a; Kumari et al., 2024; Larnder et al., 2025; Yu et al., 2025b; El-Sehrawy et al., 2026). Together, these findings suggest that menopause-associated gut microbiome remodeling may contribute to both the increased burden of chronic disease and the growing need for long-term pharmacotherapy after menopause. One large study found that postmenopausal gut microbiomes were more similar to men’s than to those of premenopausal women (Peters et al., 2022).

The reciprocal relationship also operates in the opposite direction, whereby the gut microbiome contributes to estrogen homeostasis through the estrobolome. The estrobolome refers to gut microbial genes encoding microbial enzymes specifically involved in estrogen metabolism, which can deconjugate estrogens in the gut and promote reabsorption through enterohepatic circulation (Plottel and Blaser, 2011). Through this process, the gut microbiome contributes to maintenance of circulating estrogen concentrations despite hepatic conjugation and biliary excretion. The functional capacity of the estrobolome varies substantially among individuals and is shaped by differences in gut microbial composition as well as host and environmental factors, including age, diet, medication exposure, antibiotic use, obesity, and other lifestyle factors (Hu et al., 2023; Larnder et al., 2025). Consequently, alterations in gut microbial communities may influence the extent of estrogen deconjugation and enterohepatic recirculation, potentially contributing to interindividual differences in circulating estrogen concentrations.

Although the magnitude and clinical significance of these reciprocal interactions between estrogen and the gut microbiome remain an active area of investigation, accumulating evidence suggests that menopause represents a critical period during which disruption of this host-microbiome relationship may have important physiologic and pharmacologic consequences. Declining estrogen concentrations during menopause may disrupt this reciprocal relationship, resulting in endocrine and microbial changes that reinforce one another (Baker et al., 2017; Mou et al., 2026). Because many therapeutic agents undergo the same hepatic conjugation and enterohepatic recirculation pathways as endogenous estrogens, menopause-associated alterations in this host-microbiome endocrine axis provide a biologically plausible framework for understanding interindividual variability in drug response. The following section reviews the principal mechanisms through which these microbiome-mediated processes influence medication efficacy and toxicity.

## 4 Gut microbiome-mediated mechanisms influencing drug response

The same microbial enzymes involved in estrogen metabolism also participate in the metabolism of numerous medications. Many therapeutic agents undergo hepatic glucuronidation before biliary excretion, after which microbial β-glucuronidases, a key estrogen enzyme, can regenerate active parent compounds within the intestinal lumen. Thus, the estrobolome serves as a biologic intersection between endocrine physiology and pharmacology. In particular, menopause-associated changes in microbial β-glucuronidase activity have the potential to influence not only estrogen homeostasis but also the disposition, efficacy, and toxicity of glucuronidated medications (Hu et al., 2023). This shared metabolic pathway provides the strongest mechanistic rationale for investigating how menopause-associated alterations in the gut microbiome may contribute to variability in drug response.

The gut microbiome influences drug response through multiple mechanisms (**Figure 2**). These include regulation of enterohepatic recirculation, direct microbial metabolism of medications, modification of bile acid metabolism, production of bioactive metabolites, and maintenance of intestinal barrier integrity and immune homeostasis. Collectively, these pathways influence drug absorption, bioavailability, pharmacokinetics, pharmacodynamics, therapeutic efficacy, and adverse drug reactions. However, these mechanisms differ substantially in the strength of available evidence. Microbial β-glucuronidase-mediated enterohepatic recirculation is supported by the most robust experimental and clinical data, whereas the remaining mechanisms represent emerging pathways whose relevance to menopause-associated variability in drug response requires further investigation.

**Figure 2:**
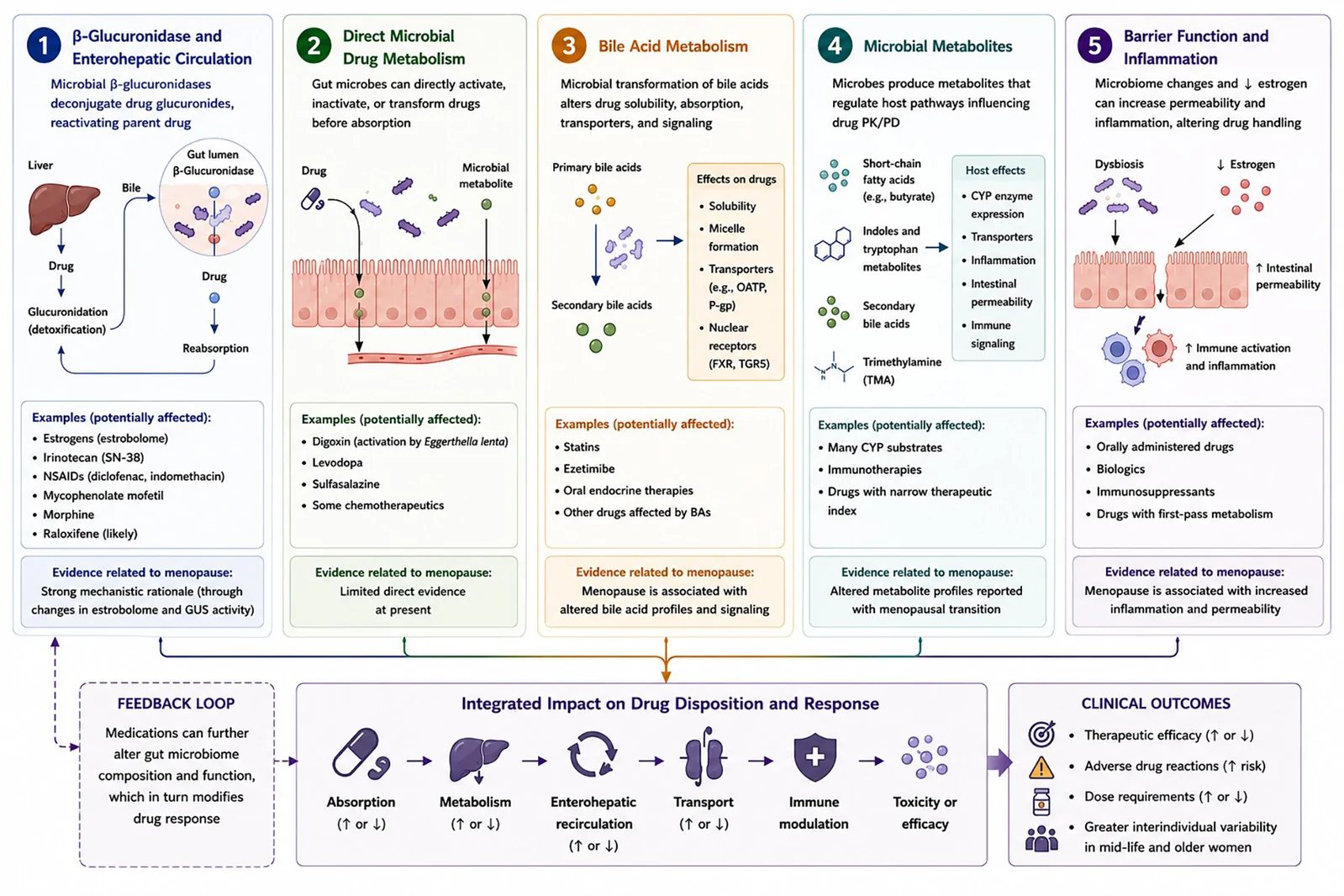
Proposed mechanisms by which the gut microbiome may influence drug response during menopause. The gut microbiome influences drug pharmacokinetics and pharmacodynamics through multiple interconnected mechanisms. These include (1) microbial β-glucuronidase activity and enterohepatic recirculation, (2) direct microbial metabolism of medications, (3) modification of bile acid metabolism and signaling, (4) production of bioactive microbial metabolites that regulate host signaling pathways, and (5) modulation of intestinal barrier integrity, inflammation, and immune function. Collectively, these pathways may alter drug absorption, metabolism, bioavailability, efficacy, toxicity, and adverse drug reactions. Among these mechanisms, β-glucuronidase-mediated enterohepatic recirculation currently has the strongest experimental and clinical support, whereas the contributions of the remaining pathways to menopause-associated variability in drug response require further investigation.

### 4.1 Microbial β-glucuronidase activity and enterohepatic recirculation

Among the most extensively studied enzymes, microbial β-glucuronidases hydrolyze glucuronide-conjugated estrogens that have undergone hepatic phase II metabolism (Ervin et al., 2019). Following hepatic glucuronidation, estrogens are secreted into bile and delivered to the intestinal lumen as inactive conjugates. Microbial β-glucuronidases remove the glucuronic acid moiety, regenerating biologically active estrogens that can subsequently be reabsorbed through enterohepatic circulation. This process contributes to maintenance of circulating estrogen concentrations and represents an important mechanism through which the gut microbiome participates in endocrine regulation and homeostasis.

Many drugs undergo hepatic glucuronidation as part of phase II metabolism, producing inactive, water-soluble glucuronide conjugates that are excreted into bile. Within the intestine, bacterial β-glucuronidases can hydrolyze these conjugates, regenerating the active parent compound or metabolite and promoting enterohepatic recirculation (Gao et al., 2022). Depending on the drug, this process may prolong drug exposure, alter therapeutic efficacy, or increase toxicity. The best-characterized example is irinotecan, for which microbial reactivation of the inactive glucuronide metabolite (SN-38G) produces active SN-38 within the colon, leading to dose-limiting diarrhea and intestinal injury (Mathijssen et al., 2001; Tobin et al., 2003). Similar mechanisms have been described for nonsteroidal anti-inflammatory drugs (NSAID), mycophenolate mofetil, morphine, and raloxifene (Carmody and Turnbaugh, 2014; Dashnyam et al., 2018; Dikeocha et al., 2022; Gao et al., 2022; Drevland et al., 2025). Although direct clinical studies remain limited, this pathway provides the most compelling mechanistic link between menopause-associated microbiome changes and variability in drug response.

### 4.2 Direct microbial metabolism of drugs

In addition to modifying glucuronidated compounds, the gut microbiota possesses an extensive repertoire of enzymes capable of directly metabolizing xenobiotics before absorption or excretion. These microbial enzymes can activate, inactivate, or transform medications with altered pharmacologic properties (Zimmermann et al., 2019). Unlike hepatic metabolism, microbial biotransformation occurs within the intestinal lumen and varies according to microbial composition. Well-established examples include reduction of digoxin (Haiser et al., 2013; Kumar et al., 2018), bacterial activation of the prodrug sulfasalazine in the colon (Peppercorn and Goldman, 1972), and microbial metabolism of levodopa (Maini Rekdal et al., 2019), each of which alters drug bioavailability or therapeutic efficacy. While menopause has been associated with changes in gut microbial composition and function, little is known regarding how these changes influence microbial xenobiotic metabolism. Nevertheless, alterations in the abundance or activity of drug-metabolizing microorganisms represent a plausible source of interindividual variability in medication response during midlife and beyond.

### 4.3 Altered bile acid metabolism

The gut microbiome regulates the composition and signaling properties of the bile acid pool through microbial deconjugation, oxidation, epimerization, dehydroxylation, and other enzymatic transformations, several of which convert host-derived primary bile acids into secondary bile acids (Ridlon and Gaskins, 2024). Beyond their role in lipid digestion and absorption, bile acids function as signaling molecules through receptors including the nuclear receptor farnesoid X receptor and the membrane G protein-coupled bile acid receptor 1, commonly known as TGR5, thereby influencing glucose and lipid metabolism, inflammatory signaling, and intestinal barrier integrity (Fiorucci and Distrutti, 2015; Wahlström et al., 2016; Ramírez-Pérez et al., 2017; Song et al., 2025). By contributing to micellar solubilization and activating nuclear-receptor signaling pathways, changes in the bile acid pool can influence the intestinal absorption and enterohepatic disposition of some orally administered drugs and regulate the expression of hepatic and intestinal transporters and xenobiotic-metabolizing enzymes (Pavlović et al., 2018). Reviews of the emerging literature suggest that menopause is associated with alterations in gut microbial composition and microbial metabolic pathways, including bile acid metabolism (Yu et al., 2025a). However, whether these changes contribute to clinically meaningful differences in drug pharmacokinetics or pharmacodynamics remains largely unknown (Mohanty et al., 2024; Yu et al., 2025a). Menopause-associated remodeling of microbial bile acid metabolism therefore represents a biologically plausible mechanism through which drug absorption, disposition, efficacy, or toxicity could be modified, but direct clinical evidence supporting this relationship remains limited.

### 4.4 Microbial metabolites and host signaling

Beyond direct drug metabolism, the gut microbiome produces numerous bioactive metabolites that regulate host physiology and may indirectly influence medication response (Nicholson et al., 2012; Krautkramer et al., 2021). Short-chain fatty acids, indole derivatives, secondary bile acids, trimethylamine, and other microbial metabolites affect immune function, inflammatory signaling, intestinal epithelial integrity, and hepatic metabolism through interactions with host receptors and signaling pathways (Lavelle and Sokol, 2020; Krautkramer et al., 2021). These metabolites can influence the expression and activity of cytochrome P450 enzymes, membrane transporters, and nuclear receptors involved in xenobiotic metabolism and disposition, thereby potentially affecting both pharmacokinetics and pharmacodynamics (Li et al., 2016; Krautkramer et al., 2021). Menopause has been associated with alterations in microbial metabolic activity, including changes in short-chain fatty acid production and bile acid profiles, although findings remain somewhat inconsistent across studies (Yu et al., 2025a). As understanding of the microbial metabolome expands, these signaling molecules may prove to be important mediators linking menopause-associated microbiome changes with variability in therapeutic response (Krautkramer et al., 2021). Altered microbial metabolite production may contribute to variability in pharmacodynamics, although direct evidence remains limited.

### 4.5 Intestinal barrier function, inflammation, and immune modulation

The gut microbiome is essential for maintaining intestinal epithelial integrity and regulating mucosal and systemic immune responses (Belkaid and Hand, 2014; Lavelle and Sokol, 2020). Dysbiosis can disrupt epithelial tight junctions, increase intestinal permeability, and promote chronic low-grade inflammation through translocation of microbial products such as lipopolysaccharide (Chelakkot et al., 2018). Declining estrogen concentrations during menopause have independently been associated with alterations in intestinal barrier function, increased inflammatory signaling, and changes in immune regulation, suggesting that hormonal and microbial factors may act synergistically to influence host physiology (Baker et al., 2017; Yu et al., 2025a). Although these mechanisms do not directly metabolize medications, they may influence therapeutic response indirectly by altering intestinal absorption, hepatic drug metabolism, immune-mediated pharmacologic effects, and susceptibility to adverse drug reactions (Li et al., 2016). While direct evidence linking menopause-associated intestinal barrier dysfunction to altered pharmacokinetics remains limited, this pathway represents an important emerging area for future pharmacomicrobiomics research. Changes in intestinal permeability and immune regulation represent promising but largely unexplored mechanisms affecting medication response.

## 5 Clinical implications and knowledge gaps

The emerging understanding of interactions among menopause, the gut microbiome, and drug metabolism highlights the limitations of traditional prescribing approaches that rely primarily on demographic and clinical characteristics. Although current evidence is insufficient to support microbiome-guided prescribing in routine clinical practice, recognition of the host–microbiome endocrine axis provides a new framework for understanding interindividual variability in medication efficacy and toxicity among midlife and older women.

Despite growing interest in pharmacomicrobiomics, important knowledge gaps remain. Clinical drug trials rarely assess or report menopausal status using standardized criteria, and direct comparisons of drug response across premenopausal, perimenopausal, and postmenopausal women are uncommon. Likewise, characterization of the gut microbiome has generally emphasized taxonomic composition rather than microbial functional capacity. Important modifiers, such as concomitant medication use, menopausal hormone therapy, diet, obesity, and co-morbid conditions, are also infrequently integrated into study design or analysis. Consequently, our understanding of how menopause-associated changes in the gut microbiome influence pharmacokinetics and pharmacodynamics remains limited. The contributions of non-bacterial components of the microbiome, including the virome, mycobiome, and archaeome, to menopause-associated changes in drug response also remain largely unexplored. Moreover, reliance on fecal sampling, which primarily reflects distal colonic luminal communities and incompletely captures mucosa-associated and region-specific gastrointestinal microbiota, may limit characterization of microbial functions relevant to drug disposition.

## 6 Future research priorities

Traditional approaches to drug prescribing may not fully account for the complex biologic factors contributing to interindividual variability in drug response. Increasing evidence suggests that age, diet, smoking, obesity, comorbidities, polypharmacy, hormonal status, and the gut microbiome interact in ways that influence both pharmacokinetics and pharmacodynamics. Achieving precision pharmacotherapy for menopausal women will require integrated multi-omics studies combining clinical, hormonal, microbiome, and pharmacologic data. Large, longitudinal, multi-center datasets integrating clinical, hormonal, microbiome, and pharmacologic data can facilitate systems-level analyses capable of identifying complex interactions that cannot be detected in smaller studies. Future clinical studies should include longitudinal sampling across the menopausal transition with repeated hormonal, microbiome, medication, and clinical outcome measurements. In addition, future studies should assess both taxonomic composition and microbial functional capacity, including β-glucuronidase activity, xenobiotic-metabolizing enzymes, bile acid profiles, microbial metabolites, and metatranscriptomic or metabolomic activity. Indeed, because microbial functional capacity may be more informative than taxonomy alone for predicting medication response, functional profiling should become a priority in pharmacomicrobiomics studies.

Given the renewed recognition of the favorable benefit–risk profile of menopausal hormone therapy for appropriately selected women (Mendoza et al., 2022; The North American Menopause Society, 2022) and its increasing use in clinical practice, future studies should evaluate whether the route of administration, formulation, timing of initiation, and duration of therapy influence gut microbial composition, microbial functional capacity, or microbiome-mediated drug response. Lastly, beyond observational studies, intervention trials will be necessary to determine whether modifying the gut microbiome through dietary interventions, probiotics, prebiotics, postbiotics, or selective microbial enzyme inhibitors can improve medication efficacy or reduce adverse drug reactions in menopausal women. Such studies would provide the strongest evidence for a causal role of the microbiome in pharmacotherapy.

## 7 Conclusion

Menopause represents a period of profound physiologic change during which alterations in estrogen concentrations remodel the gut microbiome, while the gut microbiome, in turn, contributes to hormone homeostasis and influences drug disposition through multiple interconnected mechanisms. As women age, both chronic disease burden and medication use increase, making this life stage particularly relevant to understanding sources of interindividual variability in drug response. Although the evidence is strongest for menopause-associated changes in the gut microbiome and for microbiome-mediated effects on drug metabolism, direct evidence linking menopause-related microbiome remodeling to clinically actionable differences in medication efficacy, toxicity, or dosing remains limited. Addressing these knowledge gaps will require integrated studies of hormonal status, microbial functional capacity, and pharmacologic outcomes. Such research has the potential to advance precision pharmacotherapy for midlife and older women by identifying novel determinants of medication response and informing more individualized therapeutic strategies.
